# Supplementary material for: Pregnant Women’s Exposure to Household Air Pollution in Rural Bangladesh: A Feasibility Study for Poriborton: The CHANge Trial
Source: Int J Environ Res Public Health. 2022 Jan 2;19(1):482. doi: 10.3390/ijerph19010482 (PMC8744871; doi:10.3390/ijerph19010482)

# Pregnant Women's Exposure to Household Air Pollution in Rural Bangladesh: A Feasibility Study for Poriborton: The CHANge Trial

**Jonathan Thornburg [1,*], Sajia Islam [2], Sk Masum Billah [2,3], Brianna Chan [1], Michelle McCombs [1], Maggie Abbott [1], Ashraful Alam [3] and Camille Raynes-Greenow [3]**

[1] RTI International, Technology-Advancement-Commercialization, 3040 Cornwallis Road, Research Triangle Park, NC, 27707; USA; bchan.contractor@rti.org (B.C.); mmccombs@rti.org (M.M.); meabbott@ncsu.edu (M.A.)

[2] Maternal and Child Health Division, International Centre for Diarrheal Disease Research, Bangladesh (icddr,b) Mohakhali, Dhaka 1212, Bangladesh; sajiaislam@icddrb.org (S.I.); billah@icddrb.org (S.M.B.)

[3] The University of Sydney, Sydney School of Public Health. Edward Ford Building (A27), Camperdown, NSW 2006, Australia; Neeloy.alam@sydney.edu.au (A.A.); camille.raynes-greenow@sydney.edu.au (C.R.-G.)

* Correspondence: jwt@rti.org

**Table S1.** Comparison of mean (SD) hourly $PM_{2.5}$ concentrations (μg/m$^3$) measured during baseline and intervention. Bold concentrations are higher than the comparison value.

| Hour of Day | Baseline | Intervention | p-value | Likely Cause of the Difference or Lack Thereof |
|---|---|---|---|---|
| 0 | 24.5 (44.8) | **90.6 (53.5)** | <0.0001 | Ambient $PM_{2.5}$ |
| 1 | 20.3 (119.7) | **88.3 (45.2)** | <0.0001 | Ambient $PM_{2.5}$ |
| 2 | 37.6 (148.5) | **76.5 (38.0)** | <0.0001 | Ambient $PM_{2.5}$ |
| 3 | 20.5 (37.2) | **77.4 (38.3)** | <0.0001 | Ambient $PM_{2.5}$ |
| 4 | 14.7 (23.3) | **74.9 (36.9)** | <0.0001 | Ambient $PM_{2.5}$ |
| 5 | 33.8 (170.5) | **82.0 (46.6)** | <0.0001 | Ambient $PM_{2.5}$ |
| 6 | 101.3 (290.7) | 96.5 (53.2) | 0.541 | |
| 7 | **231.1 (497.6)** | 140.0 (138.6) | <0.0001 | Traditional Stove |
| 8 | 131.6 (373.3) | 125.0 (83.0) | 0.456 | |
| 9 | 61.5 (330.6) | **106.3 (128.4)** | <0.0001 | Stove Stacking, likely |
| 10 | 73.0 (358.9) | 78.7 (94.5) | 0.556 | |
| 11 | 37.8 (153.1) | **75.4 (118.7)** | <0.0001 | Ambient $PM_{2.5}$ |
| 12 | 68.4 (252.5) | 78.4 (127.0) | 0.174 | |
| 13 | **107.8 (348.0)** | 62.8 (67.8) | <0.0001 | Traditional Stove |
| 14 | **86.3 (360.6)** | 59.4 (53.9) | <0.001 | Traditional Stove |
| 15 | 40.3 (199.6) | **69.3 (171.9)** | <0.0001 | Ambient $PM_{2.5}$ |
| 16 | **120.9 (365.0)** | 76.4 (71.1) | <0.0001 | Traditional Stove |
| 17 | 146.2 (421.2) | 139.3 (182.5) | 0.561 | Traditional Stove & Stove Stacking |
| 18 | 64.5 (221.9) | **159.0 (167.3)** | <0.0001 | Stove stacking for heat, Kerosene lamps |
| 19 | 59.5 (136.1) | **132.4 (73.2)** | <0.0001 | Stove stacking for heat, Kerosene lamps |
| 20 | 31.5 (55.6) | **140.7 (85.9)** | <0.0001 | Stove stacking for heat, Kerosene lamps |
| 21 | 42.1 (107.1) | **109.0 (52.5)** | <0.0001 | Stove stacking for heat, Kerosene lamps |
| 22 | 29.7 (42.8) | **95.1 (51.3)** | <0.0001 | Ambient $PM_{2.5}$ |
| 23 | 26.5 (39.6) | **89.3 (42.1)** | <0.0001 | Ambient $PM_{2.5}$ |

**Table S2.** shows the hourly average $PM_{2.5}$ concentrations for both baseline and intervention conditions corrected for background $PM_{2.5}$. The correction factors applied were 13.7 and 58.4 μg/m$^3$ for baseline and intervention, respectively.

<u>Baseline Background PM2.5 Correction Equation</u>

$$C_{t,cor} = C_{t,u} - C_{bkg} + 20.8$$

Where:

- $C_{t,cor}$ is the corrected hourly average baseline concentration.
- $C_{t.u}$ is the measured (uncorrected) hourly average baseline concentration.
- $C_{bkg}$ is the average of the measured (uncorrected) hourly average baseline concentration of hours 0, 1, 2, 3, 4, 5, 11, 15, 18, 19, 20, 21, 22, and 23. These hours corresponded to the times when cooking was not performed as reported by participant questionnaire responses. $C_{bkg} = 34.5$
- 20.8 is a constant. The value was determined by setting $C_{t,cor}$ equal to 1 for the minimum value of $C_{t,u}$ (14.7, at 0400 hours).

<u>Intervention Background PM2.5 Correction Equation</u>

$$C_{t,cor} = C_{t,u} - C_{bkg} + 20.8$$

Where:

- $C_{t,cor}$ is the corrected hourly average intervention concentration.
- $C_{t.u}$ is the measured (uncorrected) hourly average intervention concentration.
- $C_{bkg}$ is the average of the measured (uncorrected) hourly average intervention concentration of hours 0, 1, 2, 3, 4, 5, 10, 11, 12, 13, 14, 15, 16, 21, 22, and 23. These hours corresponded

to the times when cooking was not performed as reported by participant questionnaire responses. $C_{bkg} = 34.5$

- 21.8 is a constant. The value was determined by setting $C_{t,cor}$ equal to 1 for the minimum value of $C_{t,u}$ (59.4, at 1400 hours).

Selection of $C_{t,cor}$ equal to 1 when calculating the constant was required to avoid negative concentrations. This approach is reasonable because the difference between the baseline and intervention constants is 1 (20.8 versus 21.8). The small difference means the application of this simple background correction equation did not influence the interpretation of the PM2.5 concentration data.

**Table S2.** Hourly mean (SD) $PM_{2.5}$ concentrations corrected for background $PM_{2.5}$ levels.

| Hour of Day | Baseline (ug/m$^3$) | Intervention (ug/m$^3$) | Traditional Background Corrected | Intervention Background Corrected |
|---|---|---|---|---|
| 0 | 24.5 | 90.6 | 10.8 (23.1) | 32.2 (19.4) |
| 1 | 20.3 | 88.3 | 6.6 (13.2) | 29.9 (15.4) |
| 2 | 37.6 | 76.5 | 23.9 (91.1) | 18.1 (9.1) |
| 3 | 20.5 | 77.4 | 6.8 (13.3) | 19.0 (9.4) |
| 4 | 14.7 | 74.9 | 1.0 (1.7) | 16.5 (8.2) |
| 5 | 33.8 | 82 | 20.1 (110.8) | 23.6 (13.5) |
| 6 | 101.3 | 96.5 | 87.6 (273.5) | 38.1 (21.3) |

| | | | | |
|---|---|---|---|---|
| 7 | 231.1 | 140 | 217.4 (505.7) | 81.6 (82.8) |
| 8 | 131.6 | 125 | 117.9 (311.5) | 66.6 (43.5) |
| 9 | 61.5 | 106.3 | 47.8 (185.6) | 47.9 (59.9) |
| 10 | 73 | 78.7 | 59.3 (121.8) | 20.3 (25.2) |
| 11 | 37.8 | 75.4 | 24.1 (106.4) | 17.0 (27.9) |
| 12 | 68.4 | 78.4 | 54.7 (218.8) | 20.0 (33.4) |
| 13 | 107.8 | 62.8 | 94.1 (330.3) | 4.4 (4.9) |
| 14 | 86.3 | 59.4 | 72.6 (333.0) | 1.0 (0.9) |
| 15 | 40.3 | 69.3 | 26.6 (142.9) | 10.9 (27.9) |
| 16 | 120.9 | 76.4 | 107.2 (332.5) | 18.0 (17.1) |
| 17 | 146.2 | 139.3 | 132.5 (400.6) | 80.9 (109.2) |
| 18 | 64.5 | 159 | 50.8 (190.5) | 100.6 (108.6) |
| 19 | 59.5 | 132.4 | 45.8 (113.3) | 74.0 (41.3) |
| 20 | 31.5 | 140.7 | 17.8 (31.6) | 82.3 (51.2) |
| 21 | 42.1 | 109 | 28.4 (78.7) | 50.6 (25.0) |

| | | | | |
|---|---|---|---|---|
| 22 | 29.7 | 95.1 | 16.0 (24.9) | 36.7 (18.0) |
| 23 | 26.5 | 89.3 | 12.8 (20.6) | 30.9 (14.9) |

**Figure S1**. Location of rice mills and brick kilns within the study area that were in operation during the LPG cookstove intervention phase.

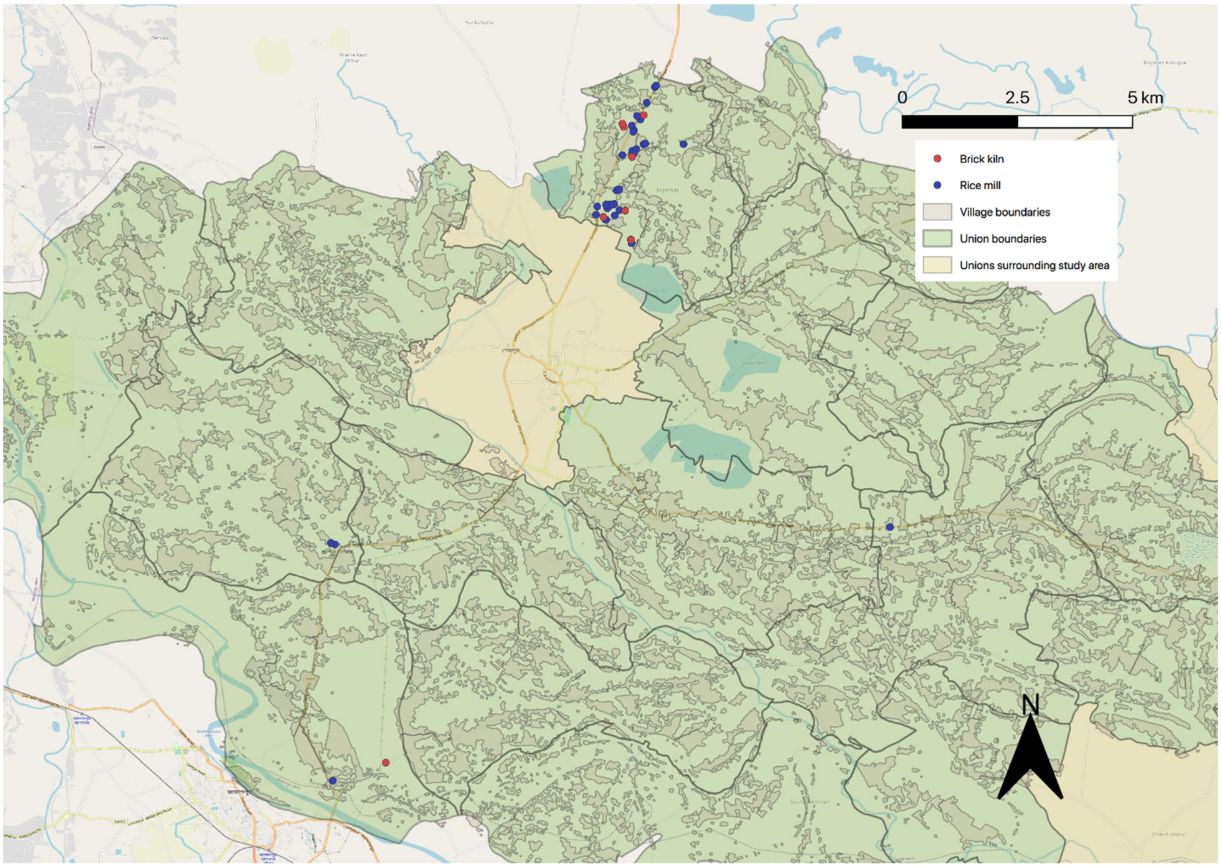

Supplement: Supplementary file 1 [file ijerph-19-00482-s001.zip › ijerph-1500111-supplementary.pdf]
